# Pri-miR-124 rs531564 polymorphism and colorectal cancer risk

**DOI:** 10.1038/srep14818

**Published:** 2015-10-01

**Authors:** Xue-ren Gao, Hui-ping Wang, Shu-long Zhang, Ming-xi Wang, Zhan-sheng Zhu

**Affiliations:** 1 Department of Microbiology and Immunology, Medical School of Southeast University, Nanjing, China; 2 Department of Genetics, Xuzhou Medical College, Xuzhou, China; 3 Department of General Surgery, Zhongda Hospital, Medical School of Southeast University, Nanjing, China; 4 Department of Medical Oncology, The First Affiliated Hospital of Bengbu Medical College, Bengbu, China; 5 Department of Pathology, Xuzhou Medical College, Xuzhou, China

## Abstract

MiR-124 functions as a tumor suppressor and plays an important role in tumorigenesis. A common polymorphism (rs531564, C>G) in the pri-miR-124 has been recently studied in connection with cancer risk. The aim of the present study was to investigate the association between pri-miR-124 rs531564 polymorphism and the risk and clinicopathological characteristics of colorectal cancer (CRC). Two case-control studies involving 900 CRC patients and 1110 cancer-free controls showed that pri-miR-124 rs531564 polymorphism was significantly associated with the decreased risk of CRC in Xuzhou population [GG vs. CC: OR = 0.25, 95%CI = 0.09–0.67, *P* = 0.003; (CG+GG) vs. CC: OR = 0.73, 95%CI = 0.56–0.94, *P* = 0.01; GG vs. (CC+CG): OR = 0.27, 95%CI = 0.10–0.70, *P* = 0.004; G vs. C: OR = 0.70, 95%CI = 0.56–0.89, *P* = 0.003], Bengbu population [GG vs. CC: OR = 0.20, 95%CI = 0.04–0.90, *P* = 0.02; GG vs. (CC+CG): OR = 0.21, 95%CI = 0.05–0.95, *P* = 0.03; G vs. C: OR = 0.72, 95%CI = 0.54–0.98, *P* = 0.03] and pooled population [GG vs. CC: OR = 0.26, 95%CI = 0.11–0.59, *P*<0.001; (CG+GG) vs. CC: OR = 0.76, 95%CI = 0.62–0.93, *P* = 0.008; GG vs. (CC+CG): OR = 0.27, 95%CI = 0.12–0.62, *P *< 0.001; G vs. C: OR = 0.71, 95%CI = 0.59–0.85, *P*<0.001]. Additionally, pri-miR-124 rs531564 polymorphism was significantly associated with the decreased risk of poor differentiation and lymph node metastasis of CRC. Our results suggest that pri-miR-124 rs531564 polymorphism may be a genetic modifier for developing CRC. However, further studies are needed to validate our findings.

Colorectal cancer (CRC) is a major public health problem and a leading cause for human deaths in the world^1^. Although the pathogenesis of CRC remains largely unknown, a number of studies have demonstrated that CRC is a complex disease that results from environmental and genetic factors. Host genetic factors may play an important role in individual susceptibility to CRC^2^,^3^.

MicroRNAs (miRNAs) are a class of endogenous, small, noncoding RNAs that modulate the activity of specific mRNA targets and play vital roles in a wide range of physiologic and pathologic processes. Increasing evidence showed that deregulation of miRNAs expression was involved in tumor initiation and progression. MiR-124 was initially confirmed to be a brain-enriched miRNA which was necessary for embryonic neuronal differentiation^4^,^5^. Recently, miR-124 has been found to be a potential tumor suppressor, which is epigenetically silenced in various types of cancer and regulates the biological behaviors of cancer cell by targeting several important genes, including rho-kinase 2 (ROCK2), enhancer of the zeste homologue 2 (EZH2), sphingosine kinase 1 (SPHK1) and the androgen receptor^6^,^7^,^8^,^9^.

Single nucleotide polymorphisms (SNPs) are a type of common genetic variation associated with disease susceptibility^10^. The functional polymorphisms in the miRNA gene may affect the expression or function of miRNA, and have attracted increasing attention. A common polymorphism (rs531564, C > G) in the pri-miR-124 has been identified and shown to influence the expression of mature miR-124^11^. In addition, pri-miR-124 rs531564 polymorphism was reported to be significantly correlated with the risk of various cancers, including cervical cancer and esophageal squamous cell carcinoma (ESCC)^12^,^13^,^14^. Thus, we hypothesized that the potentially functional polymorphism also contributed to the risk of CRC.

In the current study, we firstly conducted two independent case-control studies in Chinese population to investigate the associations, and then examined the clinicopathological characteristics of CRC for different genotypes.

## Results

### Pri-miR-124 rs531564 polymorphism and CRC risk

The selected characteristics of enrolled subjects are listed in Table 1. No significant difference was found in distributions of age and gender. Association of the pri-miR-124 rs531564 polymorphism with CRC risk was shown in Table 2. In Xuzhou population, the pri-miR-124 rs531564 polymorphism was significantly associated with the decreased risk of CRC [GG vs. CC: OR = 0.25, 95%CI = 0.09–0.67, *P* = 0.003; (CG+GG) vs. CC: OR = 0.73, 95%CI = 0.56–0.94, *P* = 0.01; GG vs. (CC+CG): OR = 0.27, 95%CI = 0.10–0.70, *P* = 0.004; G vs. C: OR = 0.70, 95%CI = 0.56–0.89, *P* = 0.003]. Similar results were also observed in Bengbu population [GG vs. CC: OR = 0.20, 95%CI = 0.04–0.90, *P* = 0.02; GG vs. (CC+CG): OR = 0.21, 95%CI = 0.05–0.95, *P* = 0.03; G vs. C: OR = 0.72, 95%CI = 0.54–0.98, *P* = 0.03] and pooled population [GG vs. CC: OR = 0.26, 95%CI = 0.11–0.59, *P *< 0.001; (CG + GG) vs. CC: OR = 0.76, 95%CI = 0.62–0.93, *P* = 0.008; GG vs. (CC + CG): OR = 0.27, 95%CI = 0.12–0.62, *P *< 0.001; G vs. C: OR = 0.71, 95%CI = 0.59–0.85, *P *< 0.001].

### Clinicopathological characteristics of CRC for different genotypes

The association between pri-miR-124 rs531564 polymorphism and clinicopathological characteristic of CRC patients was conducted in two independent studies (Table 3). Significant associations were found in status of differentiation and lymph node metastasis. In CRC patients from Xuzhou city, the pri-miR-124 rs531564 polymorphism was associated with the decreased risk of poor differentiation [(CG+GG) vs. CC: OR = 0.67, 95%CI = 0.45–0.99, *P* = 0.05; G vs. C: OR = 0.69, 95%CI = 0.48–0.99, *P* = 0.04], and lymph node metastasis [CG vs. CC: OR = 0.65, 95%CI = 0.43–0.96, *P* = 0.03; (CG+GG) vs. CC: OR = 0.64, 95%CI = 0.43–0.95, *P* = 0.03; G vs. C: OR = 0.67, 95%CI = 0.47–0.96, *P* = 0.03]. In CRC patients from Bengbu city, the pri-miR-124 rs531564 polymorphism was also associated with the decreased risk of poor differentiation [(CG + GG) vs. CC: OR = 0.56, 95%CI = 0.33–0.98, *P* = 0.04; G vs. C: OR = 0.58, 95%CI = 0.34–0.96, *P* = 0.03], and lymph node metastasis [(CG+GG) vs. CC: OR = 0.58, 95%CI = 0.34–0.97, *P* = 0.04; G vs. C: OR = 0.59, 95%CI = 0.37–0.96, *P* = 0.03]. Furthermore, these associations of pri-miR-124 rs531564 polymorphism with the decreased risk of poor differentiation [(CG+GG) vs. CC: OR = 0.66, 95%CI = 0.48–0.90, *P* = 0.01; G vs. C: OR = 0.65, 95%CI = 0.48–0.87, *P* = 0.003] and lymph node metastasis [CG vs. CC: OR = 0.71, 95%CI = 0.51–0.98, *P* = 0.04; (CG + GG) vs. CC: OR = 0.64, 95%CI = 0.47–0.88, *P* = 0.006; G vs. C: OR = 0.62, 95%CI = 0.46–0.82, *P *< 0.001] were also observed in pooled patients.

## Discussion

Similar to the protein-coding genes, miRNAs are transcribed for the majority by RNA polymerase II as long primary miRNA (pri-miRNAs) transcripts with stem-loop structure^15^. The pri-miRNAs are processed to produce miRNA precursors (pre-miRNAs) and then further cleaved into mature miRNAs which act mainly through annealing to 3′ untranslated regions (3′UTRs) of gene transcripts, leading to inhibition of further steps of gene expression. MiR-124 is a highly conserved miRNA and plays an important role in neural processes, from nervous system development to normal neuronal cell function^16^. Nowadays, miR-124 has been reported to be a potential tumor suppressor and an independent prognostic marker in many cancers, including colorectal cancer, prostate cancer, and nasopharyngeal carcinoma^17^,^18^,^19^. The miR-124 gene is located in 8p23 and has an identified polymorphism (rs531564) which affects miR-124 expression^11^. The associations of rs531564 polymorphism and cancer risk have been reported; however, the results of previous studies are controversial. Most studies showed that rs531564 polymorphism was associated with decreased risk of cancer, such as cervical cancer and esophageal squamous cell carcinoma; however, one study indicated no association between rs531564 polymorphism and cancer risk including esophageal cancer, which was probably due to difference in genotyping method, cancer type or source of controls^12^,^13^,^14^,^20^.

In two independent case-control studies from Xuzhou population (577 CRC patients and 690 healthy controls) and Bengbu population (323 CRC cases and 420 healthy subjects), we found that pri-miR-124 rs531564 polymorphism was significantly associated with the decreased risk of CRC, which is similar to the results of the recent meta-analysis^21^. Analysis based on bioinformatics and gene expression showed that the G allele in the rs531564 polymorphism changed the formation of a ring-shaped structure in miR-124, which was beneficial to the increase of the amount of mature miR-124^11^. Therefore, we speculated that the role of pri-miR-124 rs531564 polymorphism in CRC susceptibility was associated with the stability of the pri-miRNA, or the efficiency of processing of pri-miRNA into mature miRNA. Furthermore, our results showed that pri-miR-124 rs531564 polymorphism was significantly associated with clinicopathological characteristics of CRC patients, including poor differentiation and lymph node metastasis. To the best of our knowledge, this is the first molecular epidemiological study to investigate the associations of pri-miR-124 rs531564 polymorphism with the risk and clinicopathological characteristics of CRC. However, we could not determine the interaction between pri-miR-124 rs531564 polymorphism and potential risk factors, such as drinking, red meat consumption and body mass index, because of a lack of enough individual data in case-control studies.

Taken together, our findings suggest that pri-miR-124 rs531564 polymorphism contributes to the decreased risk of CRC, poor differentiation and lymph node metastasis in Chinese population, possibly by affecting miR-124 expression. However, further research in this field is warranted to complete understanding of carcinogenesis associated with the pri-miR-124 rs531564 polymorphism.

## Materials and Methods

### Study population

Two independent case-control studies were conducted in unrelated ethnic Han Chinese. Briefly, 577 CRC patients and 690 age and gender frequency-matched healthy controls were recruited from the Affiliated Hospital of Xuzhou Medical College between July 2013 and December 2014, and 323 CRC cases and 420 cancer-free controls were enrolled from December 2013 to January 2015 in the Affiliated Hospital of Bengbu Medical College. Patients were confirmed by routine histopathological examination and received no chemotherapy or radiation therapy before surgery. Control subjects were without any previous cancer diagnosis and selected from a pool of controls by normal physical examination. Demographic and clinicopathological data were collected from medical records and histopathology reports. Written informed consent was obtained from all patients and healthy control subjects before study entry. The study design and protocol were approved by the ethics committee of The Affiliated Hospital of Bengbu Medical College and Xuzhou Medical College, China. Methods were carried out in accordance with the approved guidelines.

### DNA extraction and genotyping

The genomic DNA of each subject was isolated from peripheral blood samples using genomic DNA extraction kit (Qiagen) according to the manufacturer’s protocol. DNA fragments containing rs531564 were amplified with the following primers (forward: 5-TCTTCTACCCACCCCTCTTCC-3; reverse: 5-AATCTGCACACACAAGCACTC-3). The PCR products were sequenced in forward direction. The SNP genotypes were determined by DNAMAN and BLAST.

### Statistical Analysis

Differences in general characteristics were examined using Student’s *t*-test or χ^2^ test. The Hardy-Weinberg equilibrium (HWE) was tested by a goodness-of-fit χ^2^ test to compare the expected genotype frequencies with observed genotype frequencies in cancer-free controls. Logistic regression was used to analyze the association between the rs531564 polymorphism and the risk and clinicopathological characteristics of CRC, adjusted by confounding factors including age, gender, family history of cancer, tumor site, differentiation, TNM and/or lymph node metastasis when appropriate. In Table 3, all the comparisons are done using the second characteristic as reference. *P *< 0.05 was used as the criterion of statistical significance. All statistical analyses were implemented in the SPSS statistical software (version 19.0).

## Additional Information

**How to cite this article**: Gao, X.- *et al.* Pri-miR-124 rs531564 polymorphism and colorectal cancer risk. *Sci. Rep.* **5**, 14818; doi: 10.1038/srep14818 (2015).

## Figures and Tables

**Table 1:** General characteristics of study population.

**Characteristics**	**Xuzhou population**		**Bengbu population**		**Pooled population**	
	**Cases, n (%)**	**Controls, n (%)**	**Cases, n (%)**	**Controls, n (%)**	**Cases, n (%)**	**Controls, n (%)**
Age						
≤60 years	277 (48.0)	334 (48.4)	174 (53.9)	235 (56.0)	451 (50.1)	569 (51.3)
>60 years	300 (52.0)	356 (51.6)	149 (46.1)	185 (44.0)	449 (49.9)	541 (48.7)
Gender						
Male	325 (56.3)	399 (57.8)	187 (57.9)	226 (53.8)	512 (56.9)	625 (56.3)
Female	252 (43.7)	291 (42.2)	136 (42.1)	194 (46.2)	388 (43.1)	485 (43.7)
Family history of cancer						
Yes	71 (12.3)	63 (9.1)	33 (10.2)	34 (8.1)	104 (11.6)	97 (8.7)
No	506 (87.7)	627 (90.9)	290 (89.8)	386 (91.9)	796 (88.4)	1013 (91.3)
Tumor site						
Rectum	311 (53.9)		180 (55.7)		491 (54.6)	
Colon	266 (46.1)		143 (44.3)		409 (45.4)	
Differentiation						
Poor	289 (50.1)		131 (40.6)		420 (46.7)	
Good + Moderate	288 (49.9)		192 (59.4)		480 (53.3)	
Lymph node metastasis						
Yes	301 (52.2)		165 (51.1)		466 (51.8)	
No	276 (47.8)		158 (48.9)		434 (48.2)	
TNM pathological stage						
I and II	248 (43.0)		152 (47.1)		400 (44.4)	
III and IV	329 (57.0)		171 (52.9)		500 (55.6)	

**Table 2:** The genotype and allele frequencies of the pri-miR-124 rs531564 polymorphism in CRC patients and controls

**Model comparison**	**Genotype/allele**	**Cases, n (%)**	**Controls, n (%)**	**OR (95% CI)**	***P*-value**
Co-dominant model	Xuzhou population	n = 577	n = 690		
	CC	446 (77.3)	492 (71.3)	Reference	
	CG	126 (21.8)	176 (25.5)	0.79 (0.61–1.02)	0.07
	GG	5 (0.9)	22 (3.2)	**0.25 (0.09**–**0.67)**	**0.003**
Dominant model	CC	446 (77.3)	492 (71.3)	Reference	
	CG+GG	131 (22.7)	198 (28.7)	**0.73 (0.56**–**0.94)**	**0.01**
Recessive model	CC+CG	572 (99.1)	668 (96.8)	Reference	
	GG	5 (0.9)	22 (3.2)	**0.27 (0.10**–**0.70)**	**0.004**
Allele model	C	1018 (88.2)	1160 (84.1)	Reference	
	G	136 (11.8)	220 (15.9)	**0.70 (0.56**–**0.89)**	**0.003**
	Bengbu population	n = 323	n = 420		
Co-dominant model	CC	247 (76.5)	298 (71.0)	Reference	
	CG	74 (22.9)	110 (26.2)	0.81 (0.58–1.14)	0.22
	GG	2 (0.6)	12 (2.9)	**0.20 (0.04**–**0.90)**	**0.02**
Dominant model	CC	247 (76.5)	298 (71.0)	Reference	
	CG+GG	76 (23.5)	122 (29.0)	0.75 (0.54–1.04)	0.09
Recessive model	CC+CG	321 (99.4)	408 (97.1)	Reference	
	GG	2 (0.6)	12 (2.9)	**0.21 (0.05**–**0.95)**	**0.03**
Allele model	C	568 (87.9)	706 (84.0)	Reference	
	G	78 (12.1)	134 (16.0)	**0.72 (0.54**–**0.98)**	**0.03**
	Pooled population	n = 900	n = 1110		
Co-dominant model	CC	693 (77.0)	790 (71.2)	Reference	
	CG	200 (22.2)	286 (25.8)	0.81 (0.66–1.00)	0.05
	GG	7 (0.8)	34 (3.1)	**0.26 (0.11**–**0.59)**	**<0.001**
Dominant model	CC	693 (77.0)	790 (71.2)	Reference	
	CG+GG	207 (23.0)	320 (28.8)	**0.76 (0.62**–**0.93)**	**0.008**
Recessive model	CC+CG	893 (99.2)	1076 (96.9)	Reference	
	GG	7 (0.8)	34 (3.1)	**0.27 (0.12**–**0.62)**	**<0.001**
Allele model	C	1586 (88.1)	1866 (84.1)	Reference	
	G	214 (11.9)	354 (15.9)	**0.71 (0.59**–**0.85)**	**<0.001**

**Table 3:** Association between pri-miR-124 rs531564 polymorphism and clinicopathological characteristic of CRC patients

		**Cases**			**CG versus CC**		**(CG+GG) versus CC**		**G versus C**	
**Characteristics**		**CC**	**CG**	**GG**	**OR (95% CI)**	***P*-value**	**OR (95% CI)**	***P*-value**	**OR (95% CI)**	***P*-value**
Xuzhou										
Age										
≤60 years		214	60	3	0.98 (0.66–1.45)	0.91	1.00 (0.67–1.47)	0.98	1.02 (0.72–1.46)	0.90
>60 years		232	66	2						
Gender										
Male		250	72	3	1.03 (0.69–1.54)	0.87	1.04 (0.70–1.54)	0.85	1.05 (0.73–1.51)	0.80
Female		196	54	2						
Family history of cancer										
Yes		56	15	0	0.94 (0.51–1.72)	0.83	0.88 (0.48–1.61)	0.67	0.87 (0.49–1.53)	0.63
No		390	111	5						
Tumor site										
Rectum		240	67	4	0.97 (0.65–1.44)	0.86	1.01 (0.68–1.49)	0.98	1.06 (0.74–1.52)	0.76
Colon		206	59	1						
Differentiation										
Poor		233	55	1	0.70 (0.47–1.05)	0.08	**0.67 (0.45**–**0.99)**	**0.05**	**0.69 (0.48**–**0.99)**	**0.04**
Good + Moderate		213	71	4						
Lymph node metastasis										
Yes		244	55	2	**0.65 (0.43**–**0.96)**	**0.03**	**0.64 (0.43**–**0.95)**	**0.03**	**0.67 (0.47**–**0.96)**	**0.03**
No		202	71	3						
TNM pathological stage										
I and II		195	50	3	0.84 (0.56–1.26)	0.41	0.87 (0.59–1.29)	0.49	0.92 (0.64–1.32)	0.65
III and IV		251	76	2						
Bengbu										
Age										
≤60 years		131	42	1	1.14 (0.68–1.93)	0.62	1.13 (0.68–1.90)	0.63	1.12 (0.70–1.81)	0.63
>60 years		116	32	1						
Gender										
Male		145	41	1	0.87 (0.51–1.46)	0.59	0.86 (0.51–1.45)	0.57	0.88 (0.55–1.42)	0.60
Female		102	33	1						
Family history of cancer										
Yes		24	9	0	1.28 (0.56–2.88)	0.56	1.24 (0.55–2.79)	0.61	1.17 (0.55–2.47)	0.68
No		223	65	2						
Tumor site										
Rectum		132	46	2	1.41 (0.83–2.40)	0.21	1.47 (0.86–2.49)	0.15	1.49 (0.91–2.43)	0.11
Colon		115	28	0						
Differentiation										
Poor		108	23	0	0.59 (0.34–1.02)	0.06	**0.56 (0.33**–**0.98)**	**0.04**	**0.58 (0.34**–**0.96)**	**0.03**
Good + Moderate		139	51	2						
Lymph node metastasis										
Yes	134		31	0	0.60 (0.36–1.02)	0.06	**0.58 (0.34**–**0.97)**	**0.04**	**0.59 (0.37**–**0.96)**	**0.03**
No	113		43	2						
TNM pathological stage										
I and II		110	40	2	1.45 (0.86–2.45)	0.16	1.53 (0.91–2.56)	0.11	1.53 (0.95–2.47)	0.08
III and IV		137	34	0						
Xuzhou and Bengbu										
Age										
≤60 years		345	102	4	1.05 (0.76–1.43)	0.78	1.06 (0.77–1.44)	0.73	1.06 (0.80–1.41)	0.69
>60 years		348	98	3						
Gender										
Male		395	113	4	0.97 (0.71–1.34)	0.87	0.97 (0.71–1.33)	0.85	0.98 (0.74–1.31)	0.91
Female		298	87	3						
Family history of cancer										
Yes		80	24	0	1.04 (0.64–1.69)	0.87	1.02 (0.63–1.65)	0.95	0.96 (0.61–1.51)	0.87
No		613	176	7						
Tumor site										
Rectum		372	113	6	1.12 (0.81–1.53)	0.49	1.16 (0.85–1.59)	0.34	1.19 (0.89–1.60)	0.23
Colon		321	87	1						
Differentiation										
Poor		341	78	1	0.73 (0.52–1.00)	0.05	**0.66 (0.48**–**0.90)**	**0.01**	**0.65 (0.48**–**0.87)**	**0.003**
Good + Moderate		352	122	6						
Lymph node metastasis										
Yes		378	86	2	**0.71 (0.51**–**0.98)**	**0.04**	**0.64 (0.47**–**0.88)**	**0.006**	**0.62 (0.46**–**0.82)**	**<0.001**
No		315	114	5						
TNM pathological stage										
I and II		305	90	5	1.04 (0.75–1.42)	0.83	1.07 (0.79–1.47)	0.66	1.11 (0.83–1.48)	0.47
III and IV		388	110	2						
